# A Bio-Imaging Signature as a Predictor of Clinical Outcomes in Locally Advanced Pancreatic Cancer

**DOI:** 10.3390/cancers12082016

**Published:** 2020-07-23

**Authors:** Michele Fiore, Silvia Taralli, Pasquale Trecca, Valentina Scolozzi, Luca Marinelli, Elizabeth K. A. Triumbari, Damiano Caputo, Silvia Angeletti, Massimo Ciccozzi, Alessandro Coppola, Carlo Greco, Edy Ippolito, Maria Lucia Calcagni, Roberto Coppola, Sara Ramella

**Affiliations:** 1 Radiation Oncology, Campus Bio-Medico University, 00128 Rome, Italy; p.trecca@unicampus.it (P.T.); c.greco@unicampus.it (C.G.); e.ippolito@unicampus.it (E.I.); s.ramella@unicampus.it (S.R.); 2 UOC Medicina Nucleare, Fondazione Policlinico Universitario A. Gemelli IRCCS, 00168 Rome, Italy; silvia.taralli@hotmail.it (S.T.); valentina.scolozzi1@guest.policlinicogemelli.it (V.S.); elizabethkatherine.triumbari01@icatt.it (E.K.A.T.); marialucia.calcagni@unicatt.it (M.L.C.); 3 Radiation Oncology, S. Andrea Hospital, Sapienza University of Rome, 00189 Rome, Italy; luca.marinelli@uniroma1.it; 4 Istituto di Medicina Nucleare, Università Cattolica del Sacro Cuore, 00168 Rome, Italy; 5 Department of Surgery, Campus Bio-Medico University, 00128 Rome, Italy; d.caputo@unicampus.it (D.C.); a.coppola@unicampus.it (A.C.); r.coppola@unicampus.it (R.C.); 6 Unit of Clinical Laboratory Science, Campus Bio-Medico University, 00128 Rome, Italy; s.angeletti@unicampus.it; 7 Unit of Medical Statistic and Molecular Epidemiology, Campus Bio-Medico University, 00128 Rome, Italy; m.ciccozzi@unicampus.it

**Keywords:** pancreatic cancer, chemoradiotherapy, biomarkers for predicting response, PET/CT

## Abstract

*Purpose*: To evaluate the predictive value of ^18^F-FDG PET/CT semiquantitative parameters of the primary tumour and CA 19-9 levels assessed before treatment in patients with locally advanced pancreatic cancer (LAPC). *Methods*: Among one-hundred twenty patients with LAPC treated at our institution with initial chemotherapy followed by curative chemoradiotherapy (CRT) from July 2013 to January 2019, a secondary analysis with baseline ^18^F-FDG PET/CT was conducted in fifty-eight patients. Pre-treatment CA 19-9 level and the maximum standardized uptake value (SUVmax), metabolic tumour volume (MTV) and total lesion glycolysis (TLG) of primary tumour were measured. The receiving operating characteristics (ROC) analysis was performed to define the cut-off point of SUVmax, MTV, TLG and CA 19-9 values to use in prediction of early progression (EP), local progression (LP) and overall survival (OS). Areas under the curve (AUCs) were assessed for all variables. Post-test probability was calculated to evaluate the advantage for parameters combination. *Results*: For EP, CA 19-9 level > 698 U/mL resulted the best marker to identify patient at higher risk with OR of 5.96 (95% CI, 1.66–19.47; *p* = 0.005) and a Positive Predictive Value (PPV) of 61%. For LP, the most significant parameter was TLG (OR 9.75, 95% CI, 1.64–57.87, *p* = 0.012), with PPV of 83%. For OS, the most significant parameter was MTV (OR 3.12, 95% CI, 0.9–10.83, *p* = 0.07) with PPV of 88%. Adding consecutively each of the other parameters, PPV to identify patients at risk resulted further increased (>90%). *Conclusions*: Pre-treatment CA 19-9 level, as well as MTV and TLG values of primary tumour at baseline ^18^F-FDG PET/CT and their combination, may represent significant predictors of EP, LP and OS in LAPC patients.

## 1. Introduction

Pancreatic cancer is one of the most harmful gastrointestinal cancers, whose worldwide incidence is consistently increasing, with a 5-year survival rate under 5% [1]. At the time of diagnosis, radical surgery is taken into account only in less than 20% of cases. Almost 30% of patients have a locally advanced pancreatic cancer (LAPC), the remaining patients present distant metastases [2]. Indeed, the wide local spread of the tumour to surrounding vascular structures, which is an exclusion criterion for radical surgical resection (R0), has driven endeavours to reduce tumour size and vascular invasion of the disease with the combination of chemotherapy and radiotherapy as first line treatment option. Both loco-regional and distant recurrence are common [3,4], and this suggests that local and systemic therapies have to be combined. Currently, induction chemotherapy followed by chemoradiotherapy (CRT) is the strategy considered for curative intent in patients with locally advanced unresectable cancers, or as neoadjuvant setting in borderline resectable disease [5,6]. The motivation behind neoadjuvant therapy incorporates a higher rate of R0 resections, improved local control, metastatic lymph nodes sterilization, and better selection of patients who may receive a surgical resection if appropriate [7,8]. In recent years, there have been advances in pancreatic imaging. For the assessment of resectability and tumour spread, multi-detector CT with 3-dimensional reconstruction is the best staging modality. Additionally, ^18^F-Fluorodeoxyglucose (^18^F-FDG) Positron Emission Tomography/Computed Tomography (PET/CT) plays a role in detecting distant metastases and in evaluating response to treatment. Moreover, it is also a promising tool for the definition of radiotherapy target volume. For radiotherapy planning, metabolic data obtained from ^18^F-FDG PET/CT produces a significant change in volume definition by identifying sub-volumes of treatment that may benefit from radiation dose boosting [9]. The role of ^18^F-FDG PET/CT for the optimization of treatment in pancreatic cancer is rising, not only for its implementation in radiotherapy planning, but also for its potential prognostic value. The high mortality rate of pancreatic cancer is related to local tumour progression and complications of distant metastases. In order to decrease the number of patients who do not respond to treatment or relapse, the early identification of specific pre-treatment factors as prognostic predictors may improve patient stratification and personalized treatments. The maximum standardized uptake value (SUVmax) is the most well-known and commonly used PET-derived semiquantitative parameter to measure the metabolic activity within a tumour lesion, representing the maximum value of tracer uptake in the tumour [10]. Several studies have investigated the prognostic role of SUVmax in pancreatic cancer and additionally upheld the introduction of other semiquantitative volume-based PET parameters, such as metabolic tumour volume (MTV) and total lesion glycolysis (TLG), with heterogeneity in population size, treatments and results [11,12]. Recently, a meta-analysis has shown that these metabolic parameters derived from pre-treatment ^18^F-FDG PET/CT may play a predictive role for patients with pancreatic cancer [13]. Moreover, serum carbohydrate antigen (CA) 19-9 has also been studied for its prognostic value [14]. It is the most common and validated diagnostic tumour marker and is currently being applied in clinical practice for prediction of treatment response and prognosis.

The combination of induction chemotherapy followed by chemoradiation is increasingly being used in patients with LAPC. However, there are a group of patients with early progress despite this treatment intensification, leading to an unfavourable cost-benefit ratio. Early progression can be a useful indicator in clinical practice. The question this analysis tries to answer is whether integrating humoral and functional imaging features before treatment may identify high-risk patients for progression, in whom alternative strategies could then be applied.

## 2. Materials and Methods

### 2.1. Patients

Among one-hundred and twenty patients with LAPC treated at our institution with initial chemotherapy followed by curative chemoradiotherapy (CRT) from July 2013 to January 2019, a secondary analysis from two prospective protocols with baseline ^18^F-FDG PET/CT was conducted in fifty-eight patients. Criteria for inclusion in the study were as follows: histological diagnosis of pancreatic adenocarcinoma, locally advanced non-metastatic disease, baseline ^18^F-FDG PET/CT and no history of other malignancy within 5 years, age >18 years. Patients had to show sufficient hepatic, renal, cardiac and bone marrow reserve and be able to tolerate induction chemotherapy and subsequent CRT. Patients eligible for surgery were automatically excluded from the study. Patients underwent complete pre-treatment evaluation, including clinical history and physical examination, laboratory exams (complete blood count, chemistry and CA 19-9), clinical staging based on multi-detector thin-slice multiphase contrast enhanced CT scan optimized for pancreatic imaging, laparoscopy with peritoneal washing and ^18^F-FDG PET/CT scan. Lymph nodes measuring < 1 cm in maximum transverse diameter on CT scan were considered as metastasis-positive only when characterized by increased ^18^F-FDG uptake at PET/CT scan. The lesions were judged as locally advanced unresectable tumours by our multidisciplinary gastrointestinal tumour board in accordance with NCCN Guidelines (Version 2.2018). The study was approved by the independent Ethics Committee of our university, in accordance with the principles of the Declaration of Helsinki. All patients provided written informed consent. This trial was registered at ClinicalTrials.gov with Identifier NCT02984501.

### 2.2. Treatment

All patients received a therapeutic protocol that provided initial chemotherapy followed by CRT. For induction chemotherapy, two regimens were used during the enrolment period: gemcitabine 1000 mg/m^2^ and oxaliplatin 100 mg/m^2^, and FOLFIRINOX scheme (oxaliplatin 85 mg/m^2^, irinotecan 180 mg/m^2^, leucovorin 400 mg/m^2^, fluorouracil 2400 mg/m^2^). In both schedules, chemotherapy was administered every 14 days for four doses. For patients without disease progression detected by the re-staging CT scan, chemotherapy was followed by CRT. For the study population, a four-dimensional CT simulation was performed, when possible, with respiratory training to reduce internal motion. The Clinical Target Volume (CTV) included tumour and affected nodes for a total dose of 59.4 Gy. It targets also peripancreatic lymph nodes at risk with a dose of 45 Gy with conventional fractionation. The Planning Target Volume (PTV) was obtained by expanding the CTV with a 1 cm margin in all directions to account for set-up error. Treatments were delivered with a multileaf collimator and a multifield isocentric technique (Varian Medical System). All patients received concurrent chemotherapy with gemcitabine 600 mg/m^2^ weekly. Four weeks after the completion of CRT, patients underwent clinical and imaging evaluation. Tumour response was defined in accordance with the World Health Organization (WHO) definition through CT scan. Patients were followed-up on in accordance with their clinical course every three months through a standard surveillance protocol; thereafter, and the intervals were extended to six months after two years.

### 2.3. ^18^ F-FDG PET/CT Acquisition and Analysis

**Figure 1:**
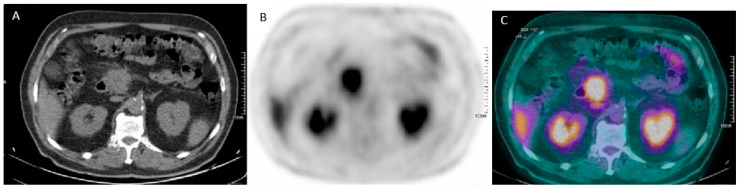
Transaxial low-dose co-registered CT (**A**), PET (**B**) and fused PET/CT (**C**) images show increased ^18^F-FDG uptake in the pancreatic tumoural lesion (**C**).

All pre-treatment PET/CT were performed at a single PET/CT Centre, 60 ± 10 min after the injection of 236 ± 45 MBq of ^18^F-FDG, according to the body mass index, from the skull base to mid-thigh region, using an integrated PET/CT device (Gemini GXL by Philips Medical System, Cleveland, OH, USA, or Biograph mCT by Siemens Healthineers, Chicago, IL, USA). All patients fasted for at least 6 h, presented blood glucose level < 150 mg/dl and were in optimal hydration state (i.v. administration of 500 mL of saline solution) at the time of tracer injection. An X-ray scout was performed to define the spatial range of acquisition, as well as a low-dose CT scan (120Kv, 50–80mA) for photon attenuation correction and fusion with PET images for anatomical localization of functional finding. PET scans were produced in 3D mode, with an acquisition time of 2–3 min per bed position, and reconstructed with iterative algorithms. Qualitative and semi-quantitative evaluation of PET images was performed by two independent nuclear medicine physicians (who were informed of all clinical and imaging information available at staging, but not made privy to the patients’ final outcome). Any focal increase in ^18^F-FDG outside normal distribution or higher than the surrounding physiological uptake at the first visual evaluation was considered an abnormal finding (see Figure 1 as representative case). Any disagreement was resolved by consensus. A 3D Volume of Interest (VOI) was manually placed over the primary tumour and the corresponding SUVmax, defined as maximum activity concentration (kBq/mL) in the VOI adjusted to the injected activity (MBq) and body weight (Kg), was measured applying the EQ∙PET reference-based quantification technology (developed by Siemens Healthineers) [15]. The MTV (expressed in cm^3^) was measured using a threshold of 40% of the SUVmax, visually checking the success of tumour delineation, with the TLG calculated as the product of the SUVmean and the MTV.

### 2.4. Statistical Analysis

All study sample characteristics were summarized with descriptive statistics. Continuous variables were reported as mean and standard deviation or median (with range). Early progression (EP) was defined temporally as a progression at the first evaluation, at 3 months from the start of treatment. EP was evaluated by CT scan. Local progression (LP) was defined as progression in any time at the site of primary disease and RECIST criteria were used. Overall survival (OS) was determined from the day of the histological diagnosis to death, or last follow-up if no event was observed. Progression-free survival (PFS) was calculated from the start of treatment to the date of progression, or to the last follow-up if no event occurred. OS and PFS curves were obtained with the Kaplan–Meier method. The receiving operating characteristics (ROC) analysis was performed to define the cut-off point of SUVmax, MTV, TLG and CA 19-9 values to use in prediction of early progression, local progression and OS. Areas under the curve (AUCs) were assessed for all variables. Post-test probability was calculated to evaluate the advantage of parameter combinations. A *p* value < 0.05 was considered statistically significant. The Med-Calc 11.6.1.0 statistical package (MedCalc Software, Mariakerke, Belgium) was used for all statistical analysis.

## 3. Results

### 3.1. Patients

From July 2013 to January 2019 fifty-eight patients (F:33; M:25) with locally advanced pancreatic adenocarcinoma were included in our study. Characteristics of the study population were described in Table 1. The median age was 64 years (range, 40–79). Pancreatic head was the most common site of presentation (91%), with 9% of tumours occurring in the body of pancreas. The mean serum CA 19-9 value among all patients was 1630 U/mL (range, 11 to 8945 U/mL). In our population, 10 patients (17%) did not show any increase in CA 19.9 value (<37 U/mL). Twenty patients (34.5%) had a severe obstructive jaundice at diagnosis. Jaundiced patients before treatment underwent biliary drainage. In these patients, CA19-9 values were included in the study once the jaundice was resolved, and before starting the treatment. With regard to pre-treatment ^18^F-FDG-PET/CT, the mean interval time between PET/CT and the beginning of treatment was 16 days (range, 2–40 days); the mean values of SUVmax, MTV and TLG of primary tumours were 6.6 ± 3.6, 20.4 ± 15.3 cm^3^, and 87.2 ± 79, respectively. The median follow-up for all patients was 12.8 months (range, 2.6 to 92.9 months). In one patient the first re-staging was earlier than planned for the clinical suspicion of progression. At the time of last evaluation, seventeen patients were alive. Twenty patients (34.5%) developed early progression at the first evaluation (six patients with local progression, eighteen patients with distant metastases, six patients with both). Overall, in thirty-nine patients (67%) a disease progression was reported during the follow-up period. Of these, fifteen patients experienced local progression, thirty-five patients had distant metastases, eleven patients had both. For the entire cohort of patients, the median OS and PFS were 14.2 months and 13.6 months, respectively. OS and PFS at 1 year were 71% and 54%, respectively.

### 3.2. ROC Curves and Post-Test Probability Analysis

**Figure 2:**
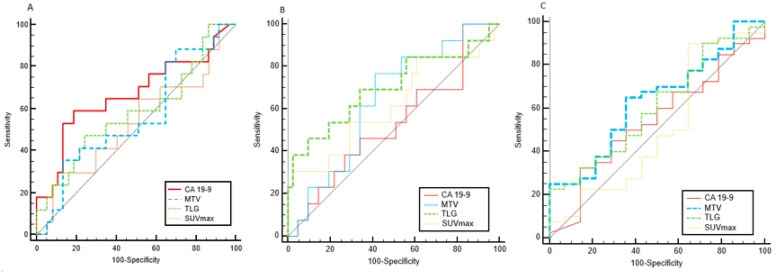
ROC curve comparison of predictors of Early Progression (EP) (**A**), Local Progression (LP) (**B**), and Overall Survival (OS) (**C**). The variables analyzed were CA 19-9 levels, the metabolic tumour volume (MTV), the total lesion glycolysis (TLG) and the maximum standardized uptake value (SUVmax) of primary tumour.

By ROC curve analysis, CA 19-9 levels, SUVmax, MTV and TLG values of primary tumour were independently tested to predict EP, LP and OS. Table 2 summarizes AUCs for each variable. ROC comparison curves of variables predicting EP, LP and OS are shown in Figure 2.

For early progression (EP), CA 19-9 level was the best marker to identify patients at higher risk with an Odds Ratio (OR) approximately six times higher when CA 19-9 was over 698 U/mL. In fact, CA 19-9 value > 698 U/mL identified EP with a Positive Predictive Value (PPV) of 61%, with a significant OR of 5.96 (95% CI, 1.66 to 19.47, *p* = 0.005). Adding other PET parameters such as MTV>32 cm^3^, SUVmax > 9 and TLG >103 consecutively, PPV was increased from 61% to 95% (Table 3).

For local progression (LP), the most significant parameter was TLG. A TLG value of over 177 indicated patients at higher risk of local progression, with a positive predictive value of 83%, and was associated with a significant OR of 9.75 (95% CI, 1.64 to 57.87, *p* = 0.012). When adding to TLG >177 the evaluation of the other two PET-derived parameters (MTV and SUVmax), when MTV is > 17 cm^3^ and SUVmax >6.5, the PPV further increased from 83% to 94% (see Table 4).

For OS, the most significant parameter was MTV. MTV over 14 cm^3^ was an indication of patients at higher risk of death with a PPV of 88% and was associated with an OR of 3.12 (95% CI, 0.9 to 10.83, *p* = 0.07). Adding to MTV >14 cm^3^ the evaluation of TLG, with TLG >167, PPV was increased from 88% to 96%; no significant difference in PPV was observed when also adding SUVmax value (cut-off: 4) (see Table 5).

## 4. Discussion

In this secondary analysis of prospective data, a bio-imaging signature of clinical outcomes after integrated chemo-radiotherapy for patients with LAPC was identified. This signature was based on the combination of ^18^F-FDG PET/CT semiquantitative parameters (SUVmax, MTV, TLG) and serum CA 19-9 level, and achieved a performance of over 90%. Serum CA 19-9 level, TLG and MTV values of the primary tumour before treatment resulted the best significant predictors of early progression, local progression and OS, respectively. Moreover, the combination of these variables is a stronger predictor of clinical outcome than the single parameter. PET-derived parameters and their prognostic role in pancreatic cancer patients treated by CRT have already been shown in several studies [13,16,17,18]. The findings of the meta-analysis of Zhu et al. [13] confirmed that PET-derived parameters at pre-treatment 18F-FDG PET/CT, despite the methodological and clinical heterogeneity observed across the included studies, might be prognostic factors in patients with pancreatic cancer, potentially useful to stratify patient risk in terms of survival and disease control: high MTV values were significant predictors of poor OS (HR 1.56, 95% CI, 1.13–2.16; *p* = 0.007) as were high TLG values (HR 1.70, 95% CI, 1.25–2.30; *p* = 0.01). Xu et al. [12] evaluated patients who received 18F-FDG PET/CT before radical pancreatectomy. In this report, TLG and MTV were significantly correlated to baseline serum CA19-9 level (*p* < 0.001 for TLG, *p* = 0.001 for MTV). Multivariate analysis showed that TLG, MTV and baseline serum CA19-9 levels were independent risk predictors for both OS and recurrence-free survival. However, studies on patients receiving chemotherapy and radiotherapy in LAPC have rarely focused on the combination of CA 19-9 levels and FDG-PET/CT parameters to predict disease progression, remaining mainly polarised on the association between PET/CT variables and survival. To the best of our knowledge, our study is the first to analyse this combination. For EP, a CA 19-9 level >698 U/mL resulted as the best marker to identify patients at higher risk with an OR of 5.96 (95%CI, 1.66–19.47). The combination of this parameter with that derived from FDG-PET/CT strengthened the positive predictive value of the marker itself, increasing from 61% to 95%. For LP, the most significant parameter was TLG>177 with a significant OR of 9.75 (95%CI, 1.64–57.87). When combined with other parameters, its positive predictive value increased from 83% to 94%. For OS, the most significant parameter was MTV with an OR of 3.12 (95% CI, 0.9–10.83). The combination of this marker with other PET-derived parameters increased the positive predictive value to 97%. Regarding metabolic parameters, in our study, no correlation between SUVmax of the tumour and EP, LP and OS was found. Therefore, MTV and TLG taken individually resulted as better predictors of progression and OS than SUVmax. In this regard, our results are in line with previous studies supporting the prognostic role of volumetric PET parameters in pancreatic cancer patients, with less concordant results on the prognostic value of SUVmax [19,20,21]. In addition, compared to MTV and TLG, adding the SUVmax of the primary tumours to the other variables leads to an increase in prediction of early and local progression, but with no evident contribution to overall survival. Furthermore, this finding seems to suggest an overall major prognostic role of the PET volumetric parameters over SUVmax. Although all of these PET parameters are used to measure the metabolic activity of a lesion, a possible explanation for the observed different prognostic values may be related to their inherent differences in methodology. Indeed, SUVmax represents the maximum voxel value of tracer concentration within the tumour (i.e., the metabolic activity value from only one voxel), whereas MTV and TLG are expressions of the whole tumour metabolic burden, also taking into consideration heterogeneous intralesional distribution of tracer uptake [10].

This study has several limitations. First, these findings are the result of a secondary analysis rather than of a prospective assignment. Second, the study population is relatively small. However, all patients had been enrolled in prospective integrated chemo-radiotherapy trials and the ^18^F-FDG PET/CT scan had been performed in each case with the same acquisition protocols and uniformly analysed by the same nuclear medicine physicians. Third, a limitation to CA 19-9 serum level evaluation in pancreatic cancer includes false negative results in Lewis negative phenotype (observed in 5–10% of patients with pancreatic cancer) [14]. In our experience, patient stratification, considering both CA 19-9 levels and ^18^F-FDG PET/CT parameters such as MTV and TLG before treatment, is able to effectively predict whether a particular patient with LAPC will or will not have early progression or disease progression during the course of treatments. In the future, the availability of this signature before treatment could allow oncologists to personalize treatments, for instance by intensifying chemotherapy as initial therapy or modifying radiation total dose, fractionation or drugs, in combination with radiotherapy, or even selecting patients for consolidation therapy. Future planned studies at our institution include the prospective validation of these results in a larger population, as well as the integration of radiomics biomarkers.

## 5. Conclusions

The combination of ^18^F-FDG PET/CT parameters and CA19-9 levels before treatment is promising for identifying patients at a higher risk of disease progression. These prognostic biomarkers might be useful for the design and development of future trials or the selection of personalized therapeutic options, possibly simplifying the management and improving the prognosis of LAPC patients.

## Figures and Tables

**Table 1:** Patients’ characteristics.

Patients’ Characteristics	*n* = 58 (%)
*Age (years)*	
Mean	64
Median	64
Range	40–79
*Gender*	
Female	33 (56.8)
Male	25 (43.2)
*Histology*	
Adenocarcinoma	58 (100)
*Stage*	
II	19 (32.7)
III	36 (67.3)
*Site of primary tumour*	
Head	53 (91)
Body	5 (9)
Istmus	0
Plus sites	0
*Initial Chemotherapy*	
Yes	58 (100)
No	0
*Combined Chemoradiotherapy*	
Yes	36 (62)
No	22 (38)

**Table 2:** Receiver operating characteristic (ROC) analysis for predictors of Early Progression (EP), Local Progression (LP) and Overall Survival (OS). The variables analyzed were CA 19-9 levels, the maximum standardized uptake value (SUVmax), the metabolic tumour volume (MTV), the total lesion glycolysis (TLG) of primary tumours. AUC, area under curve; CI, confidence interval. ns, not significant.

**Variables and EP**	**AUC**	**95% CI**
CA 19-9	0.665	0.524–0.788
SUVmax	0.558	0.416–0.693
MTV	0.534	0.393–0.671
TLG	0.579	0.437–0.712
**Variables and LP**	**AUC**	**95% CI**
CA 19-9	0.529	0.388–0.666
SUVmax	0.621	0.479–0.750
MTV	0.640	0.498–0.766
TLG	0.702	0.562–0.819
**Variables and OS**	**AUC**	**95% CI**
CA 19-9	0.543	0.402–0.679
SUVmax	0.507	0.368–0.646
MTV	0.632	0.490–0.759
TLG	0.605	0.463–0.736

**Table 3:** Predictors of Early Progression (EP). The variables analyzed were CA 19-9, the maximum standardized uptake value (SUVmax), the metabolic tumour volume (MTV), the total lesion glycolysis (TLG) of primary tumours. LR+, likelihood-ratio positive; PPV, Positive Predictive Value.

**Variables and EP**	**LR+**	**PPV (%)**
CA 19-9 >698	3.23	61
MTV >32	2.50	55
SUVmax >9	2.80	58
TLG >103	1.78	47
**Variables Combination and EP**	**PPV (%)**	
CA 19-9 >698 + MTV >32	79	
CA 19-9>698 + MTV >32+ SUVmax >9	91	
CA 19-9>698 + MTV >32+ SUVmax >9 + TLG >103	95	

**Table 4:** Predictors of Local Progression (LP). The variables analyzed were the maximum standardized uptake value (SUVmax), the metabolic tumour volume (MTV), the total lesion glycolysis (TLG) of primary tumours. LR+, likelihood-ratio positive; PPV, Positive Predictive Value.

**Variables and LP**	**LR+**	**PPV (%)**
TLG >177	14	83
MTV >17	1.65	37
SUVmax >6.5	1.87	65
**Variables Combination and LP**	**PPV (%)**	
TLG >177 plus MTV >17	89	
TLG >177 plus MTV >17 plus SUVmax >6.5	94	

**Table 5:** Predictors of Overall Survival (OS). The variables analyzed were the maximum standardized uptake value (SUVmax), the metabolic tumour volume (MTV), the total lesion glycolysis (TLG) of the primary tumours. LR+, likelihood-ratio positive; PPV, Positive Predictive Value.

**Variables and OS**	**LR+**	**PPV (%)**
MTV >14	2.03	88
TLG >167	3.51	71
SUVmax <4	1.28	48
**Variables combination and OS**	**PPV (%)**	
MTV >14 plus TLG >167	96	
MTV >14 plus TLG >167 plus SUVmax <4	97	
